# Supplementary material for: Using Task-Evoked Pupillary Response to Predict Clinical Performance during a Simulation Training
Source: Healthcare (Basel). 2023 Feb 4;11(4):455. doi: 10.3390/healthcare11040455 (PMC9956315; doi:10.3390/healthcare11040455)
Supplement: Supplementary file 1 [file healthcare-11-00455-s001.zip › healthcare-2172625-supplementary.pdf]

Table S1. Verification checklist of the intervention performance.

**Interventions**	**Correctness**
1. Assessment of consciousness, breathing, placement of Guedel cannula (unresponsive, no breathing)	
2. Request for help: call a physician	
3. Placing the patient in the supine decubitus position	
4. Introduction of the cardiac massage table	
5. Monitoring the patient: checking the rhythm on the monitor	
6. Checking the pulse	
7. Defibrillation: correct placement of paddles or electrodes apical and sternal	
8. Correct selection of shock power: shock	
9. Introduction of the cardiac massage table	
10. Starting the massage	
